# Metabolic engineering of probiotic *Escherichia coli* for cytolytic therapy of tumors

**DOI:** 10.1038/s41598-021-85372-6

**Published:** 2021-03-12

**Authors:** Chung-Jen Chiang, Po-Han Huang

**Affiliations:** Department of Medical Laboratory Science and Biotechnology, China Medical University, No. 91, Hsueh-Shih Road, Taichung, 40402 Taiwan

**Keywords:** Biotechnology, Cancer, Molecular biology, Oncology

## Abstract

Bacterial cancer therapy was developed using probiotic *Escherichia coli* Nissle 1917 (EcN) for medical intervention of colorectal cancer. EcN was armed with HlyE, a small cytotoxic protein, under the control of the araBAD promoter (P_BAD_). The intrinsic limitation of P_BAD_ for the gene expression is known to be negated by glucose and afflicted with all-or-nothing induction in host bacteria. This issue was addressed by metabolic engineering of EcN to uncouple the glucose-mediated control circuit and the L-arabinose transport-induction loop and to block L-arabinose catabolism. As a result, the reprogrammed strain (designated EcNe) enabled efficient expression of HlyE in a temporal control manner. The HlyE production was insensitive to glucose and reached a saturated level in response to L-arabinose at 30–50 μM. Moreover, the administrated EcNe exhibited tumor-specific colonization with the tumor-to-organ ratio of 10^6^:1. Equipped with HlyE, EcNe significantly caused tumor regression in mice xenografted with human colorectal cancer cells. Overall, this study proposes a new strategy for the bacteria-mediated delivery of therapeutic proteins to tumors.

## Introduction

Tumor tissues are recalcitrant to the treatment of traditional chemotherapy and radiotherapy^1^. The emerging technology known as targeted therapy facilitates selective delivery of chemotherapeutic agents involving therapeutic genes and drugs to tumor sites, which enhances the therapeutic efficacy and reduces side effects^2^. However, this approach is usually applied for proliferating cells and the therapeutic cargos have a difficulty in diffusing deep into tumor tissues^3^. The occurrence of angiogenesis in tumors develops irregular and poorly-organized blood vessels. This in turn renders the supply of oxygen in tumor vasculature insufficient, consequently leading to formation of hypoxia or anoxia regions in malignant tumors. Hypoxic cells are generally non-proliferating and exhibit high resistance to radiotherapy^4^. Apparently, there is a pressing need to rationally design delivery systems for tumor therapy.

The microenvironment surrounding tumor tissues provides a favorable niche for bacteria to inhabit. Bacteria including *Bifidobacterium*^5^, *Clostridium*^6^, *Salmonella*^7^, and *Escherichia*^8^ have been illustrated to preferentially colonize in tumors after being administrated in mice. Following bloodstream clearance mediated by inflammation, bacteria are generally entrapped in the tumor vasculature. Obligate anaerobes such as *Bifidobacterium* and *Clostridium* survive in the anoxic region. In addition, the presence of available nutrients in necrotic tumor tissues attracts facultative anaerobes like *Salmonella* and *Escherichia* to the cancerous site via chemotaxis. Consequently, they thrived in the hypoxic/necrotic regions of tumors to evade clearance by the immune system. Bacterial therapy is not new^9^, and its implementation for tumor treatment has been recently acknowledged by the advent of synthetic biology. In general, the tumor-seeking bacteria are tailored to synthesize a variety of therapeutic agents^12^. By administration locally or systemically, the engineered bacteria target tumors where they reside, replicate, and continuously produce the payloads on site. It enables in situ delivery of the produced bioactive molecules to tumor site, which improves the therapeutic efficacy.

The tumor-targeting bacteria have been genetically instructed to deliver a variety of bioactive payloads, notably involving prodrugs-converted enzymes^10^, short hairpin RNA^11^, cytokines^12^, antigens^13^, antibodies^14^, and bacterial toxins^15^. These approaches generally show encouraging results. Nevertheless, they have intrinsic limitations that most of the produced payloads are restricted to proliferating cells or/and afflicted with tumor penetration. Hemolysin appears to be a promising protein payload. It is naturally produced in bacteria and displays a pore-forming activity that lyses mammalian erythrocytes^16^. As illustrated previously, *Staphylococcus aureus* α-hemolysin (SAH) was expressed in *E. coli*^17^. Recombinant SAH was shown to penetrate into tumor tissue and eradicate cancer cells. As a result, in situ delivery of SAH by *E. coli* reduced the volume of MCF7 tumor by 41%. Like SAH, hemolysin E (HlyE) is a pore-forming protein which naturally appears in *E. coli*^18^, *S. enterica*^19^, and *Shigella flexneri*^20^. HlyE is cytotoxic to cultured mammalian cells and macrophages^21^. It causes the formation of transmembrane pore on the host cell. The damaged cell membrane in turn induces cell apoptosis. The application of the HlyE-mediated cell lysis for cancer treatment was investigated in the later work. As first exemplified by 4T1 tumor, the administration of HlyE*-*expressing *S. typhimurium* significantly decreased the tumor volume^15^.

Colorectal cancer ranks the third most common malignant tumor and is marked with a low 5-year survival rate. The number of patients who newly contracted this disease accounts for almost 10% of new cancer cases worldwide^22^. It appears necessary to further explore a potential method for medical intervention of colorectal cancer. Probiotic bacteria have emerged as the most promising chassis for living therapeutics^23^. *E. coli* Nissle 1917 (EcN) is a probiotic strain free of enterotoxins and cytotoxins and is used for the conventional treatment of various gastrointestinal illnesses^24^. EcN that produces the therapeutic proteins has been illustrated for cancer therapy in the murine tumor model. Azurin is a cytotoxic protein which induces cancer cell apoptosis. The administration of azurin-producing EcN suppressed the growth of B16 melanoma and 4T1 breast cancer while prolonged the survival of tumor-bearing mice^25^. EcN with azurin also enabled to restrain pulmonary metastasis developed by 4T1 cancer cells. EcN has innate prodrug-converting enzymes. By intratumoural injection, EcN caused a significant reduction in tumor growth and an increase in survival of the CT26 colon cell-bearing mice after prodrugs were administrated^26^. In the human tumor model, tumor growth was suppressed by engineered EcN which produced cytotoxic compounds^27^. In this study, the issue was addressed by development of bacterial cancer therapy (BCT) based on HlyE-producing EcN. To approach the goal, HlyE was expressed under the control of the *araBAD* promoter (P_*BAD*_). The strategy of metabolic engineering was applied to EcN for the temporal and spatial control of the HlyE expression. As a result, the engineered EcN preferred colonization in tumor tissues and expressed HlyE that effectively caused tumor regression in mice xenografted with human colorectal cancer cells.

## Materials and methods

### Bacterial strains and plasmids

The bacterial strain, EcNe (Δ*araBAD* Δ*araFGH* Δ*ptsG* P_EM7_::*araE lacZ::*P_BAD_*-*T7 *gene 1*), was derived from EcN and applied for this study. It was constructed in several steps as follows. First, endogenous genes of EcN were knocked out according to the previous study^28^. In brief, the passenger DNA contained FRT-*kan*-FRT flanked by two homologous regions of the *araBAD* operon and was obtained by PCR with primers (5′-atggcgattgcaattggcctc and 5′-ttactgcccttaatatgccttc). By electroporation, the PCR DNA was introduced into EcN which carried helper plasmid pKD46 for expression of λ-Red recombinase. It resulted in the replacement of the *araBAD* operon with the FRT-*kan*-FRT via homologous recombination. The inserted *kan* marker was later removed by Flp using helper plasmid pCP20^29^. In a similar way, the *araFGH* operon was knocked out with the passenger DNA which was obtained by PCR with primers (5′-atgcacaaatttactaagc and 5′-tcagacagtgcgtttcg). P1 transduction was applied to remove *ptsG*, and transducing phages were prepared from JW1087-2 (△*ptsG*::FRT-*kan*-FRT) stain. Secondly, the *araE* expression was driven by the constitutive EM7 promoter (P_*EM7*_). This was carried out by the PCR-amplification of the FRT-*kan*-FRT-P_*EM7*_ fragment from plasmid pKD-EM7 using primers 5′-gtgaggaactaaaaatcgctcctggcaggaaaaaatggttac and 5′-ggtcgacggatccccggaatatagtg-aaaaaatacgtgaac). With the act of λ-Red recombinase, P_EM7_ was substituted for the native promoter of *araE* after electroporation of the FRT-*kan*-FRT-P_EM7_ fragment. Finally, the bacterial strain was equipped with a genomic copy of T7 *gene 1* (encoding T7 RNA polymerase) under the control of P_*BAD*_.

Plasmid pBAD33-hlyE contained the *hlyE* gene of *E. coli* strain MG1655 under the control of P_*BAD*_ and was constructed as follows. By PCR, the *hlyE* DNA was amplified with primers (5′-ttatttctagatgtaaaacaggagtttcattac and 5′-gcttgaagcttctcga-gaataatcgcttatc). The PCR DNA was treated with the *Xba*I*-Hind*III digestion and incorporated into plasmid pBAD33. In addition, EcNe which carried a variant of red fluorescent protein (DsRed) under the control of the T7 promoter (P_T7_) was constructed in the following. The PCR-amplification of a DNA containing DsRed fused to P_T7_ (P_T7_-DsRed) was carried out from plasmid pRed-Coh with primers (5′-actaggatcccgcgaaattaatacgac and 5′-ctcctgctagcaaaaaacccctcaagacc). By digestion with *Bam*HI and *Nhe*I, the PCR DNA and integration plasmid pLam-LoxKm^30^ were spliced together to obtain plasmid pLam-Red. P_T7_-DsRed was integrated into EcNe genome at the prophage λ attachment site by plasmid pLam-Red.

### Production of HlyE

To produce HlyE, EcNe was transformed with plasmid pBAD33-hlyE. A single colony was picked and inoculated into a shake flask containing LB medium^31^. The bacterial growth was conducted at 37 °C and measured turbidimetrically at OD_550_. In the next day, the overnight-grown bacteria were seeded into a fresh LB medium and induced for the protein production with 30 μM L-arabinose upon OD_550_ reaching 0.3. After induction for 4 h, bacteria were harvested by centrifugation and then resuspended in 10 mM sodium phosphate buffer (PBS) at pH 7.5. Bacteria were disrupted by sonication, followed by centrifugation to collect the supernatant part. Proteins were analyzed by sulfate–polyacrylamide gel electrophoresis (SDS-PAGE) with Coomassie Blue staining and quantified using the Image Analyzer GA90000 (UVItech, England).

### Real-time digital bio-imaging

Tumor cells were seeded into a 96-well plate (5 × 10^4^ cells per well) and incubated with EcN, EcNe/HlyE, and HlyE upon reaching 80% confluence. The cell imaging was carried out by placing the plate in the multi-mode plate reader Cytation 5 (Biotek, Winooski, VT). The assay was performed at 37 °C in an atmosphere supplemented with 5% CO_2_. Images of each well were collected every 15 min over 1 h by using a 10X phase-contrast objective. Cell images were processed with GEN5 3.0 software (Biotek).

### Analysis of nuclear fragmentation

Tumor cells were seeded into a 24-well plate with a 12 mm cover slide (1 × 10^5^ cells per well) at 37 °C in an atmosphere supplemented with 5% CO_2_ overnight. After treatment with EcN or EcNe/HlyE, tumor cells were then washed twice with PBS and fixed with 2.5% paraformaldehyde for 20 min at room temperature. By washing with PBS, fixed cells were stained with 1 μg/ml diamidino-2-phenylindole (DAPI) at room temperature for 5 min. Slides were mounted with the mounting solution, followed by observing tumor cells with fluorescence microscope (Olympus IX71, Japan). Microphotographs were taken with Olympus camera.

### Cell morphology

In a similar way, tumor cells in the plate were treated with EcN and EcNe/HlyE and stained with 2.5 mg/ml propidium iodide (PI). The analysis was conducted with the multi-mode plate reader Cytation 5 (Biotek, Winooski, VT). The assay was performed at 37 °C in an atmosphere supplemented with 5% CO_2_. Images of each well were collected every 15 min over 1 h by using a 10X phase-contrast objective. Cell images were processed with GEN5 3.0 software (Biotek).

### In vitro assessment of cytotoxicity

Cell proliferation was measured using the methylthiazol tetrazolium (MTT) assay. Tumor cells in the plate were treated with EcN, EcNe/HlyE, or HlyE for 3 h and then washed twice with PBS. MTT (5 mg/ml) in PBS was added to tumor cells, followed by incubation for 4 h. In each well, the supernatant of cell culture (15 μl) was removed and replenished with the same volume of DMSO. The absorbance at 570 nm was measured with a microplate reader (BioTek EPOCH, Winooski, VT, USA).

### In vivo antitumor activity

Animal experiments exclusively complied with the Guide stipulated by the Council of Agriculture Executive Yuan and ARRIVE guidelines for the Care and Use of Laboratory Animals and were approved by the Animal Ethics Committee of China Medical University (No. 104–117-N). Specific-pathogen-free (SPF) BALB/cAnN.Cg nude male mice (4 weeks old and the body weight of 20 g) were purchased from the National Laboratory Animal Center, Taiwan. Animals were fed with drinking water containing 100 mM L-arabinose and maintained under the SPF conditions in the Animal Center at China Medical University for at least 5 days before use. The xenograft mouse models were established by implanted with the subcutaneous injection of either HT29 or SW620 cancer cells (1 × 10^7^/100 μl) on the right flank side. Bacteria were grown on LB medium for 6 h and harvested by centrifugation. Bacteria were washed by PBS, followed by centrifugation. Bacteria pellets were then resuspended in PBS for further use. EcN (10^6^/100 μl) and EcNe/HlyE (10^6^/100 μl) were administrated by two intraperitoneal or intratumoral injections on day 1 and day 5 per week or at the time when the tumor volume was > 150 mm^3^. Tumor-bearing mice were randomly assigned to 4 groups, each involving 5 mice. The body weight and tumor volume of mice were measured twice weekly. The tumor volume was calculated based on the following equation:

$${\text{Tumor}}\,{\text{volume}}\,\left( {\text{V}} \right) = {\text{length}} \times {\text{width}} \times {\text{width}}/{2}$$

At the end of experiments, mice were sacrificed to have their tumors and organs weighed. All animal experiments were repeated three times.

### Biodistribution of bacteria

Mice organs were weighed and homogenized aseptically in 3 ml of ice-cold and sterilized PBS. The homogenate was serially diluted and plated onto LB agar plates with or without chloramphenicol at 35 mg/ml. This allowed the examination of plasmid stability. Petri dishes were then incubated at 37℃ for 16 h. The colony forming unit (CFU) per gram tissue was determined by dividing bacterial by the weight of the organ.

### Histopathological study

The method of hematoxylin and eosin (H&E) stains followed the previous report^32^. Mice were anesthetized by transcardial perfusion with 4% paraformaldehyde in PBS. Tumors were blocked in longitudinal sections and processed for paraffin embedding. H&E stains were applied to tumor sections with the thickness of 5 μm, and the images were photographed using a light microscopy (Olympus, Nagano, Japan). Tumor necrosis regions were analyzed using Image J (Media Cybernetics, Bethesda, MD).

### Assessment of tumor necrosis

Tumor sections were dewaxed, rehydrated, and processed for H&E stains according to the standard histological method. Cell images were analyzed by the multi-mode plate reader Cytation 5 (Biotek, Winooski, VT) with GEN5 3.0 software (Biotek). After performing plasma disruption, organelle breakdown and cell lysis, Imag J software was applied to measure the total cross-sectional area of each tumor and the area of necrosis present in each tumor. The percentage of necrosis in each tumor section was calculated.

### Serum cytokine analysis

The analysis was performed using female C57BL/6 mice (6–8 weeks old) from National Laboratory Animal Center, Taiwan. Mice were divided into four groups (*n* = 5 mice/group). Agents, including PBS, EcNe (10^6^/100 μl), EcNe/HlyE (10^6^/100 μl), and HlyE (3.4 μg/100 μl), were administered for two consecutive days through the tail vein. Blood was collected through the retro-orbital route at 3, 6, and 24 h post the injection. The serum cytokines, TNF-α, IL-6, and interferon (IFN)-γ were quantified using ELISA kits (R&D Systems). Duplicated readings of each sample at 450 nm were analyzed following the manufacturer’s instructions.

### Statistical analysis

The statistical significance of experiments was determined by Student’s t*-*test. Results with* P* < 0.05 were considered significant.

## Results

### Design of the bacterial vector

**Figure 1:**
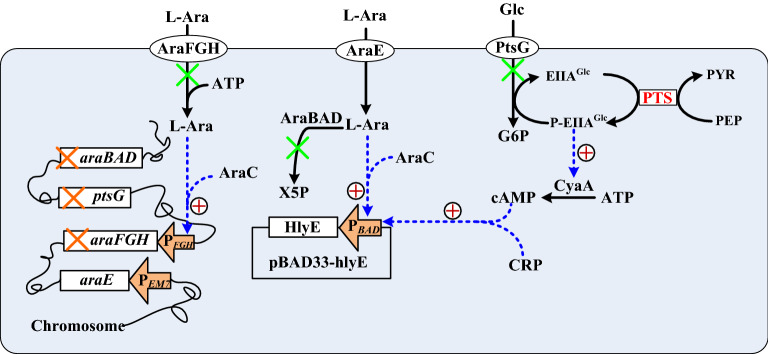
Schematic illustration of the strategy applied to rewire EcN metabolism. Shown were metabolic pathways involved in this study. Pathways of blockage and genes of deletion were marked with “X”. Through the PEP:carbohydrate phosphotransferase system (PTS), EIIA^Glc^ receives the phosphoryl group from phosphoenolpyruvate (PEP) and becomes phosphorylated (P-EIIA^Glc^) associated with the production of pyruvate (PYR). During translocation via PTS, glucose (Glc) is phosphorylated to glucose 6-phosphate (G6P) by P-EIIA^Glc^. The *araBAD* operon which consists of *araB* (encoding L-ribulokinase), *araA* (encoding L-arabinose isomerase), and *araD* (encoding L-ribulose-5-phosphate 4-epimerase) mediates conversion of L-arabinose (L-Ara) to D-xylulose 5-phosphate (X5P). P_*BAD*_ is activated (“**+**”) by regulator proteins involving AraC and CRP which complex with L-Ara and cAMP, respectively. Refer to text for more details. Notes: AraC, arabinose regulator; AraE, arabinose-proton symporter (encoded by *araE*); AraFGH, arabinose ABC transporter (encoded by *araFGH* operon); CRP, cAMP receptor protein; CyaA, adenylate cyclase; PtsG, glucose transporter (encoded by *ptsG*), P_*FGH*_, endogenous promoter of *araFGH* operon.

The concern for HlyE safety calls for an expression system with tight regulation. P_*BAD*_ is characterized with stringent regulation and exhibits a swift response to the induction of L-arabinose, which renders it useful for the expression of toxic proteins^33^. However, the expression of a P_*BAD*_-controlled gene displays the all-or-nothing induction pattern with a subsaturating level of L-arabinose^34^. This is ascribed to the “autocatalytic induction” mechanism triggered by L-arabinose which mediates the AraC-dependent activation of AraFGH for active transport of L-arabinose. Moreover, glucose interferes with the expression of P_*BAD*_ via a regulatory circuit known as catabolite repression^35^. To address these issues, EcN was reshaped by tailoring its metabolism as shown in Fig. 1. The inducer transport-induction loop was first decoupled by inactivation of *araFGH* operon, and P_EM7_-driven *araE* was employed to serve for the uptake of L-arabinose. Next, *ptsG* was knocked down to dismantle the control circuit of catabolite repression^36^. Finally, the strain was deprived of *araBAD* operon involved in the catabolic pathway of L-arabinose. This ensures the persistent inducibility of L-arabinose which is not metabolized and maintained within the bacteria. Consequently, a genetically-modified strain was obtained and designated EcNe.

**Figure 2:**
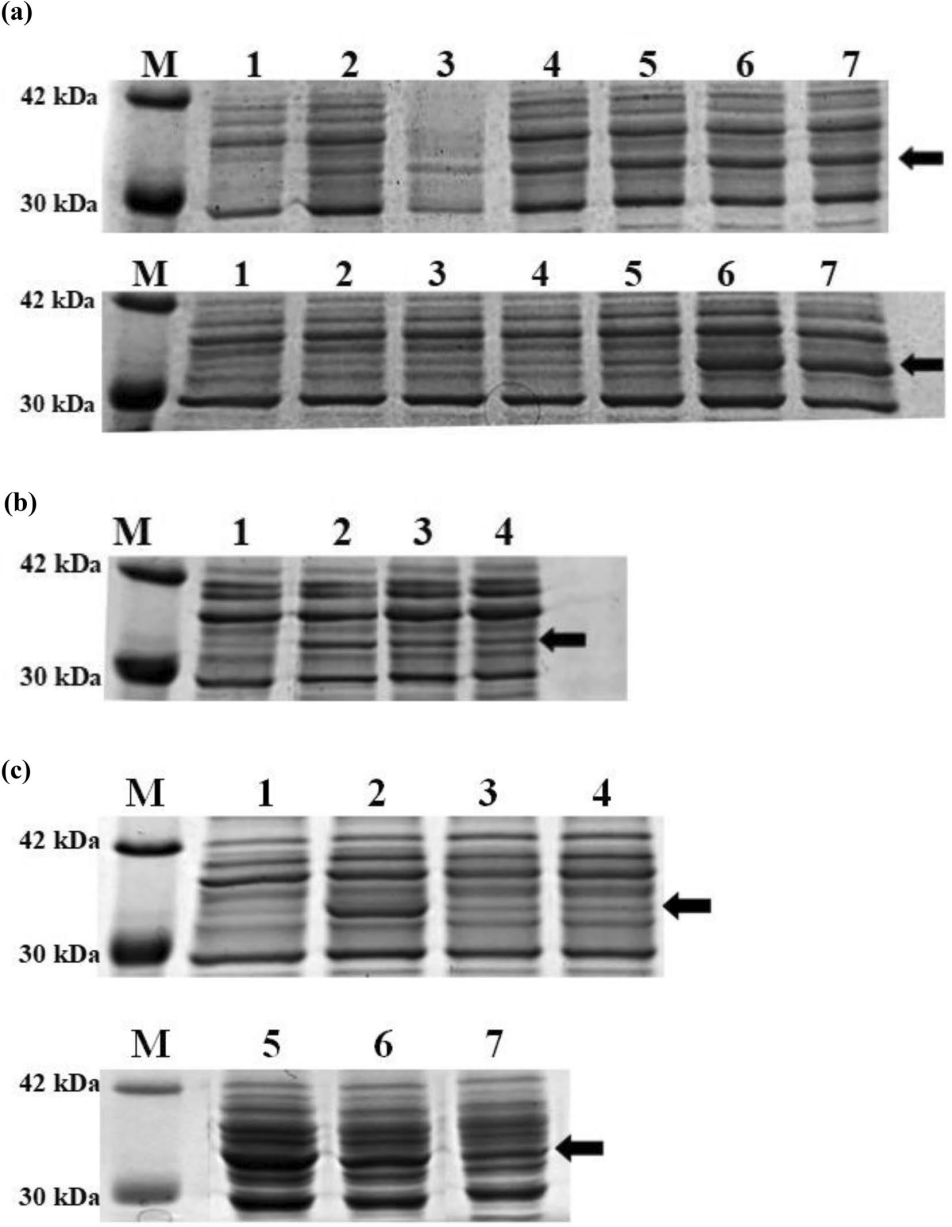
HlyE production and functional activity. (**a**)The SDS-PAGE analysis of HlyE. The production of HlyE in either EcNe/HlyE (top) or EcN/HlyE (bottom) was conducted with LB medium. The arrow indicates the location of HlyE in SDS-PAGE. Lane 1, without induction (−); lane 2, induction (+)/10 μM L-arabinose; lane 3, (+)/30 μM L-arabinose; lane 4, (+)/50 μM L-arabinose; lane 5, (+)/100 μM L-arabinose; lane 6, (+)/1 mM L-arabinose; lane 7, (+)/10 mM L-arabinose. (**b**) The effect of glucose on the HlyE production in EcNe/HlyE. EcNe/HlyE was grown on LB medium plus various amounts of glucose and induced for the HlyE production by adding 50 μM L-arabinose. Lane 1, (−)/nil glucose; lane 2, (+)/nil glucose; lane 3, (+)/glucose of 4 g/100 ml; lane 4, (+)/glucose of 10 g/100 ml. (**c**) The effect of glucose on the HlyE production in EcN/HlyE. EcN/HlyE was grown on LB medium plus various amounts of glucose and induced for the HlyE production by adding 1 mM L-arabinose. Lane 1, (−)/nil glucose; lane 2, (+)/nil glucose; lane 3, (+)/glucose of 4 g/100 ml; lane 4, (+)/glucose of 10 g/100 ml; lane 5, (+)/nil glucose; lane 6, (+)/glucose of 0.1 g/100 ml; lane 7, (+)/glucose of 0.2 g/100 ml.

EcNe which harbored plasmid pBAD33-hlyE (i.e., EcNe/HlyE) was investigated for the HlyE production. As shown in Fig. 2a,b, EcNe/HlyE produced HlyE upon induction with L-arabinose and the protein production remained unaffected in the presence of glucose. The HlyE production reached a saturated level with L-arabinose at 30–50 μM. In contrast, the HlyE production in the original strain EcN bearing pBAD33-hlyE (designated EcN/HlyE) was negated by glucose (Fig. 2c). In addition, the protein production increased with increasing L-arabinose and was saturated with L-arabinose exceeding 1 mM (Fig. 2a). In agreement with the previous report^37^, HlyE mostly accumulated within the bacterial cells. The functionality of intracellular and extracellular HlyE was further investigated by the haemolysis assay. The result showed that two forms of HlyE displayed a hemolytic zone on sheep blood agar plates (SI Fig. S1a). Furthermore, RBCs were treated with various concentrations of HlyE. The haemolysis of RBCs positively correlated to the HlyE dose (SI Fig. S1b).

### HlyE cytotoxicity

**Figure 3:**
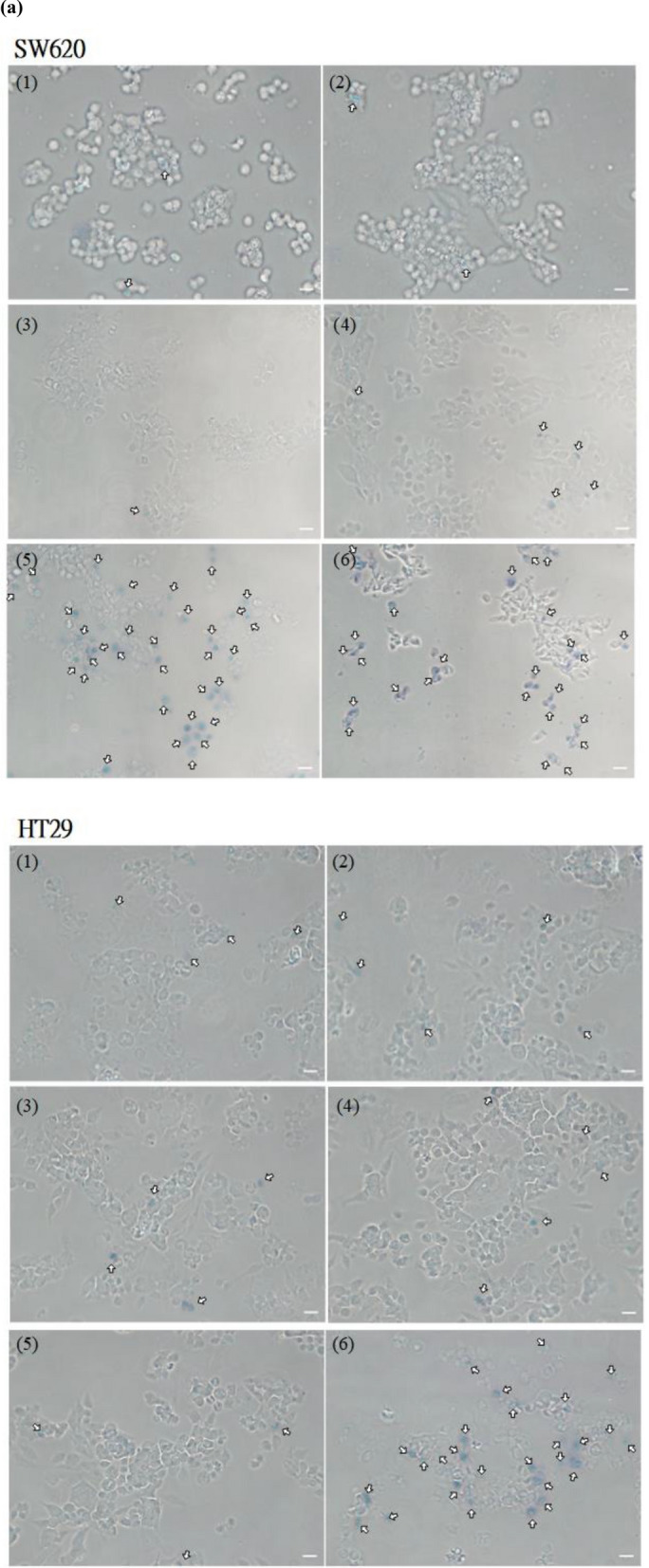

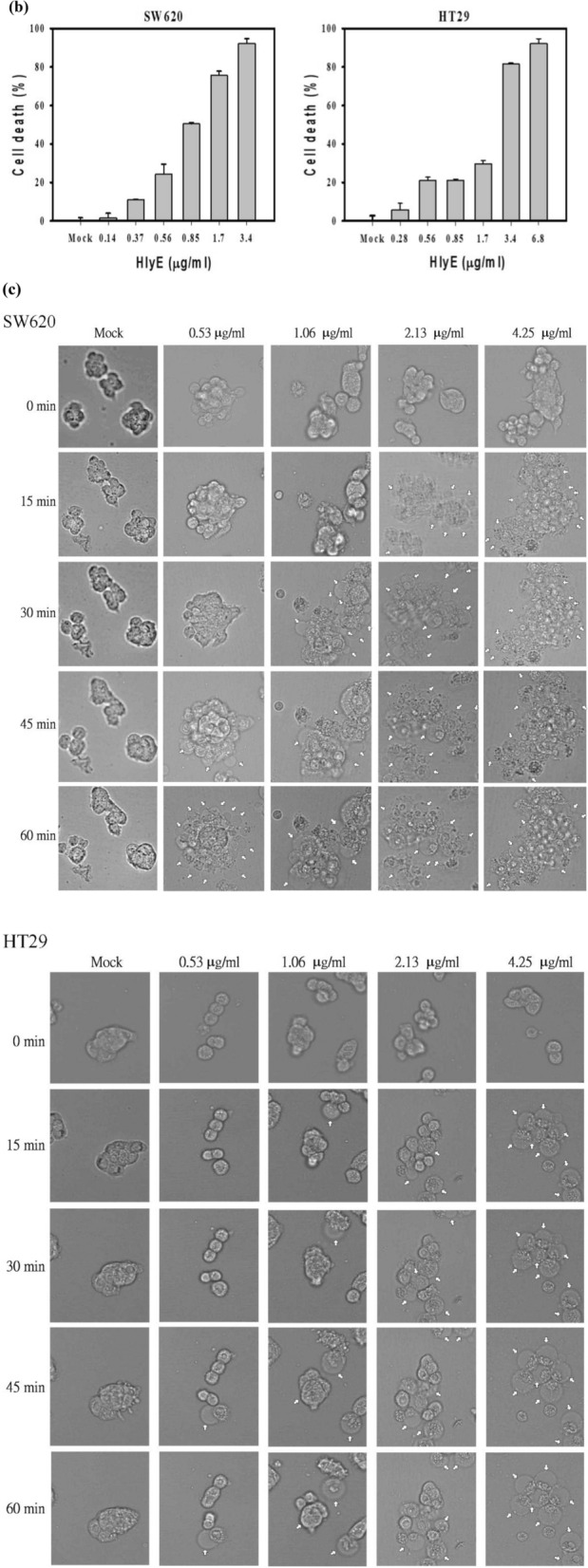
In vitro toxicity of HlyE. (**a**) Examination of cell viability by trypan blue exclusion assay. After the treatment for 30 min, cell suspension was mixed with an equal amount of 0.4% trypan blue dye. The mixture was incubated 2 min at room temperature. 1, Mock; 2, LB medium; 3, extracellular HlyE from induced EcNe/HlyE; 4, intracellular HlyE from induced EcNe/HlyE. (**b**) Examination of cell viability by MTT assay. Tumor cells were treated with HlyE from induced EcNe/HlyE for 3 h. SW620 or HT29 cells were administrated with the various levels of HlyE as indicated. (**c**) Time-lapse images of cell morphology. Images of SW620 (upper) and HT29 cells (bottom) were taken over time as indicated on the left upon the HlyE treatment. The administration dose of HlyE was shown on the top of each panel. Arrows indicate internal rearrangement of organelles and loss of membrane integrity. Cell images were processed with GEN5 3.0 software (Biotek).

The trypan blue exclusion assay provides a simple and quick examination of the HlyE-mediated effect on colorectal cancer cells. Most of SW620 and HT29 cancer cells showed a tryphan blue-stained cytoplasm after the HlyE treatment (SI Fig. S2). The cytoplasm of untreated cancer cells remained clear. The result clearly indicates that functional HlyE reduces the viability of tumor cells. The cytotoxic effect of HlyE on cell viability was further investigated by the MTT assay. Cancer cells were exposed to various concentrations of HlyE for 3 h. In general, cell viability reduced with the increasing amount of HlyE (Fig. 3a). The IC_50_ concentration of HlyE was estimated to be 0.84 and 2.36 μg/ml for SW620 and HT29 cells, respectively.

The change in cell morphology was followed along the time course by using an optical microscope. As indicated in Fig. 3b,c, SW620 and HT29 cells underwent oncosis after the HlyE treatment for 15–45 min. Cell swelling occurred in the HlyE-treated cells, followed by condensation of cellular materials at one side of cells. In general, it took 15 min for cell membranes to completely destruct after the onset of cell death.

### In vitro cytotoxicity of HlyE-producing bacteria

**Figure 4:**
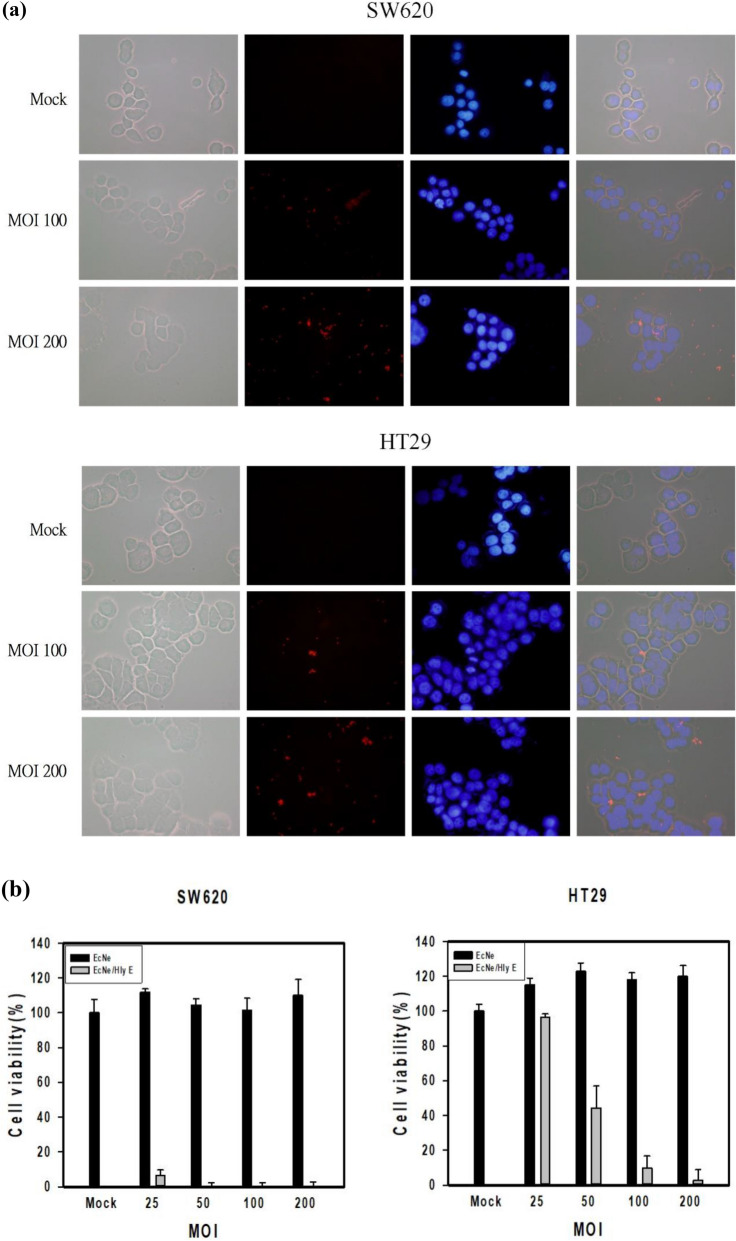

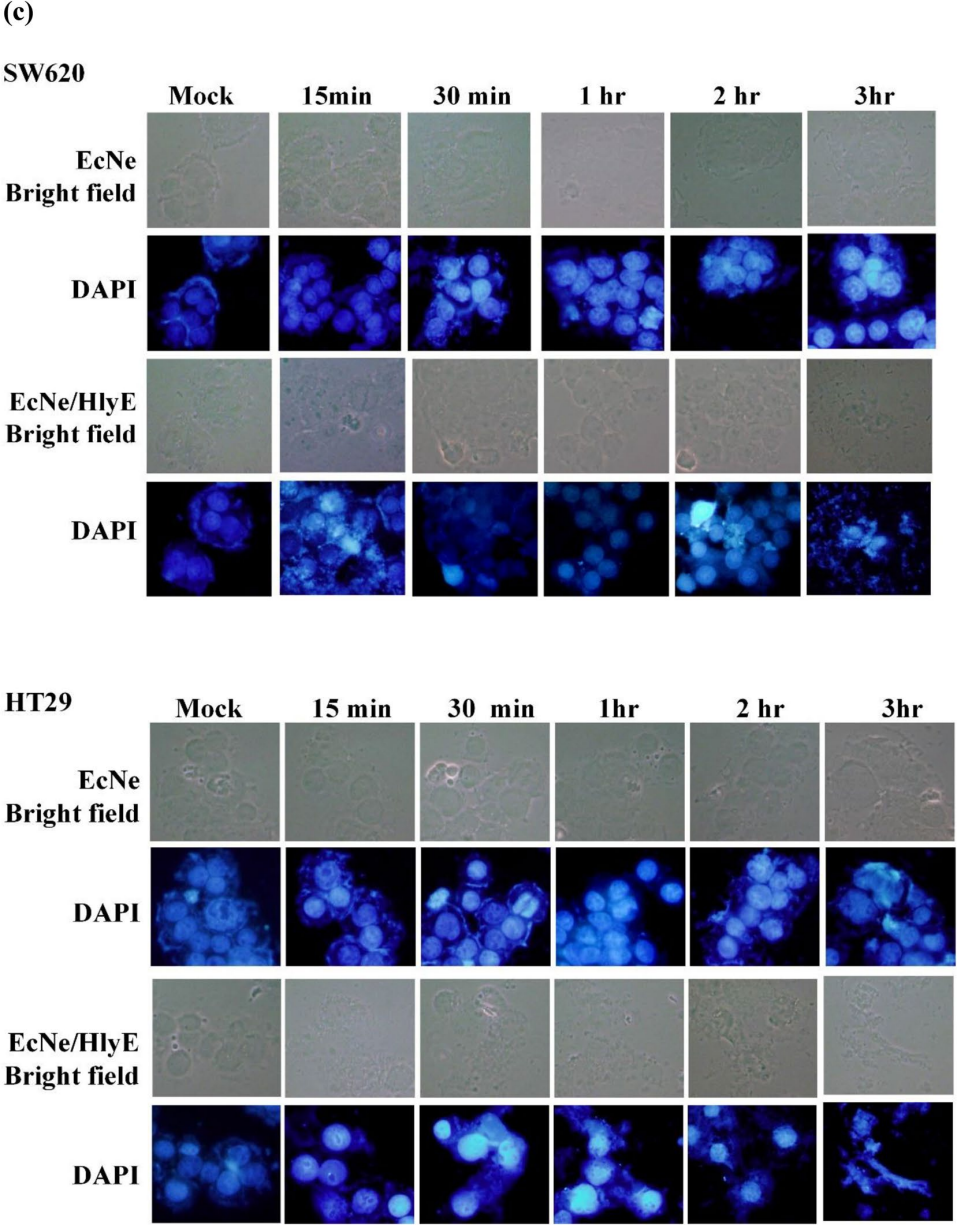

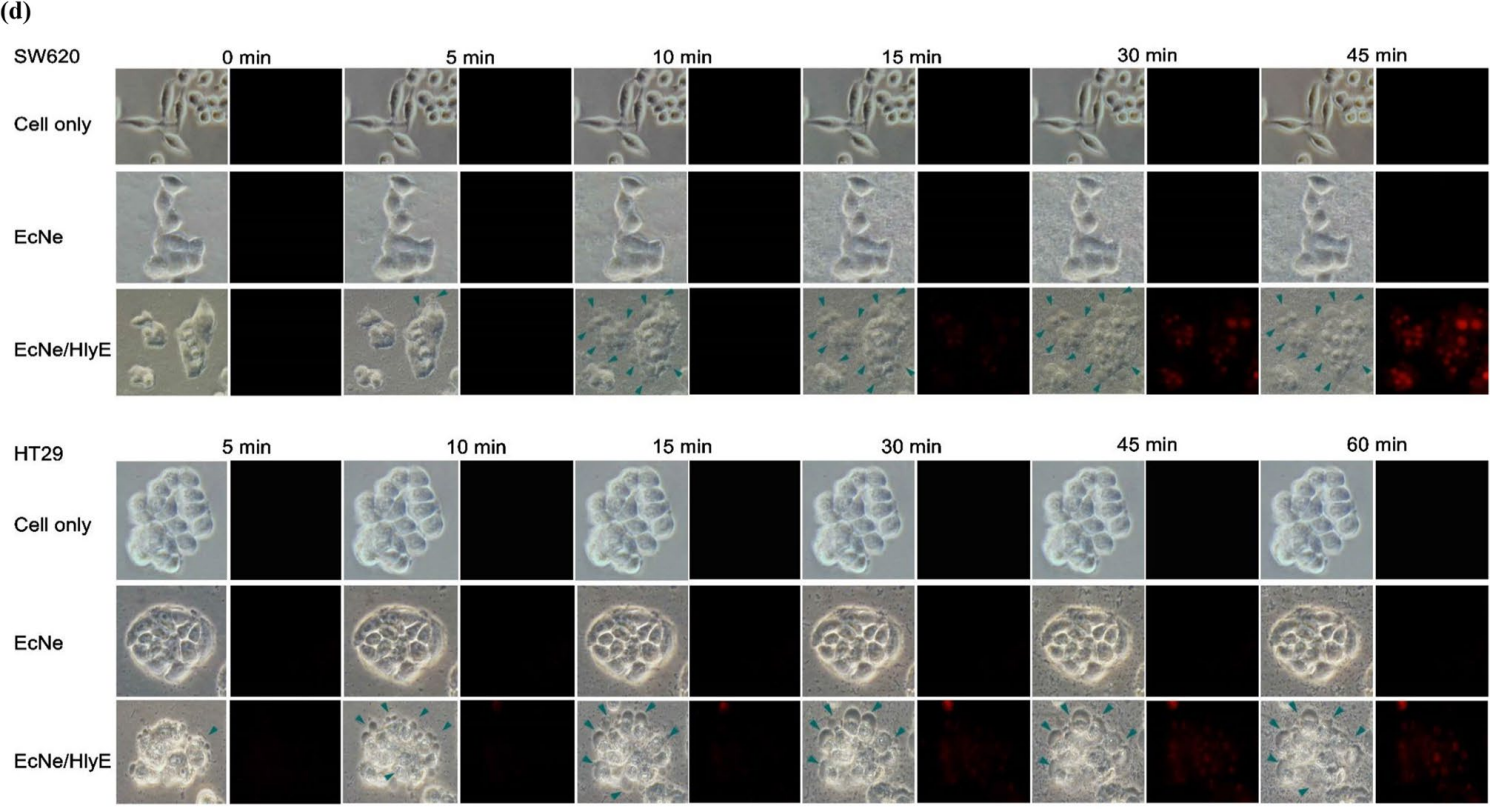
In vitro toxicity of EcNe/HlyE. (**a**) The specific attachment of EcNe to tumor cells. (**b**) Examination of cell viability by MTT assay. SW620 or HT29 cells were incubated with either EcNe or induced EcNe/HlyE at various MOI as indicated for 3 h. (**c**) Time-lapse images of cell nuclear morphology by DAPI staining. Images of SW620 and HT29 cells were taken over time as indicated on the top upon administration with either EcNe or induced EcNe/HlyE at MOI of 100. Bacteria were identified in red. (**d**) Time-lapse images of cell nuclear morphology by PI staining. Arrows indicate internal rearrangement of organelles and loss of membrane integrity. Cell images were processed with GEN5 3.0 software (Biotek).

It was informative to learn the interaction of EcNe with tumor cells. EcNe was equipped with a genomic copy of P_T7_-DsRed, resulting in EcNe/DxRed. Subsequently, either SW620 or HT29 cells were incubated with EcNe/DxRed for 1 h. As revealed by a fluorescence microscope, red signals emitted by EcNe/DxRed scattered around the cell’s periphery (Fig. 4a). It suggests that EcNe is not invasive and adheres to these colorectal cancer cells. The cytotoxic effect of EcNe/HlyE on cancer cells was further investigated. Cells were incubated with EcNe/HlyE for 3 h. EcNe free of HlyE was used for comparison. The viability of tumor cells remained unaffected by EcNe (Fig. 4b). In contrast, EcNe/HlyE displayed a strong cytotoxic activity toward tumor cells. SW620 cells were highly susceptible to EcNe/HlyE with multiplicity of infection (MOI) of 25 while the viability of HT29 cells dropped to 45% with MOI of 50.

As revealed by the PI or DAPI staining, EcNe/HlyE-treated cells exhibited the characteristic morphology of oncosis (Fig. 4c,d). The variants of oncotic cells were identified in the following. The time-lapse images showed that small membrane blebs and higher mass of cells occurred for SW620 and HT29 cells at 5 and 15 min post treatment, respectively. After the treatment for 0.5–1 h, SW620 and HT29 cells were more frequent to show larger plasmatic blebs, gradual loss of cell shape, and clumping of chromatin. In cells with the treatment for 2–3 h, it was more frequent to observe larger plasmatic blebs, lower mass of cells, significant swelling of nucleus where nuclear membrane and chromatin condensation still remained distinguishable. The homogenization of nuclear structure, cell membrane disruption with a partial extrusion of cell contents, and the morphological characteristics of necrosis were more prevalent in SW620 and HT29 cells that received the bacterial treatment for 3 and 6 h, respectively. The intensity of PI staining increased in the treated cells, indicating permeabilized cell membranes and cell death (Fig. 4d).

### Tumor regression by HlyE-producing bacteria

**Figure 5:**
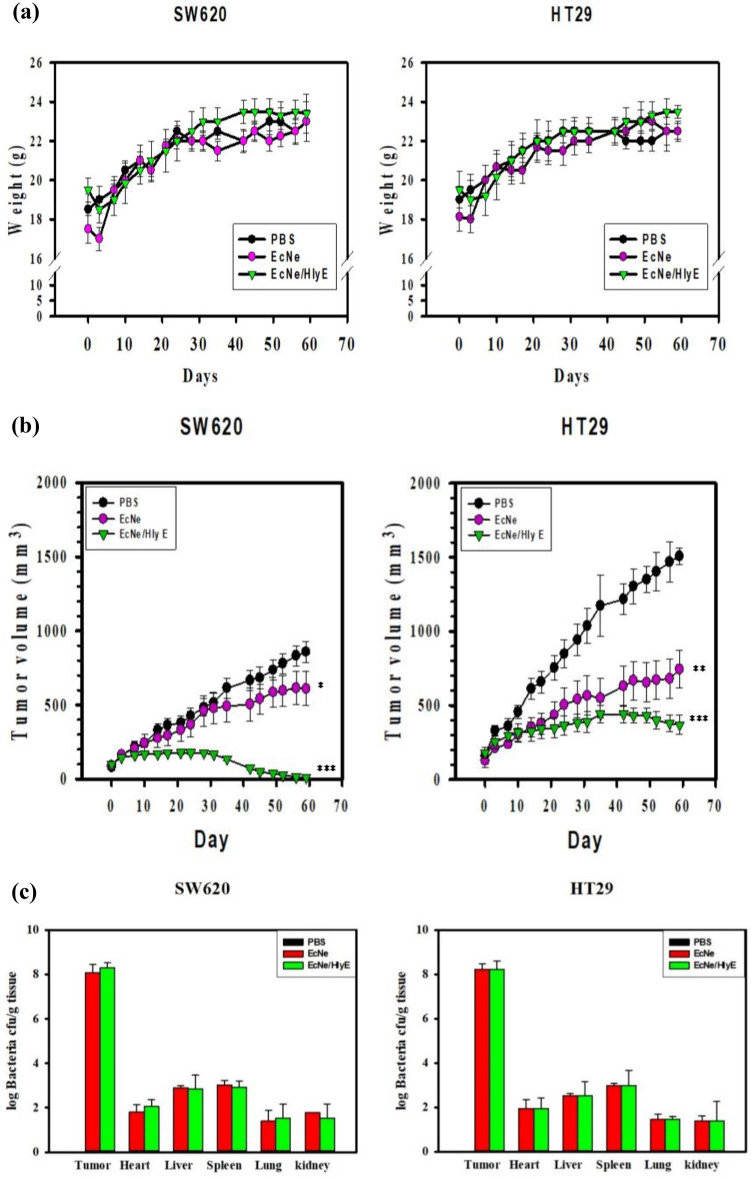
Tumor regression by EcNe/HlyE. (**a**) The change in the body weight of mice receiving intraperitoneal injection of bacteria. (**b**) Therapeutic effect of EcNe/HlyE on mice (n = 5) bearing SW620 or HT29 tumor cells. Tumor volumes (mm^3^) were estimated using external calipers, and values were expressed as means ± standard deviations (SD). The statistical significance was analyzed by Student's t-test (*p < 0.05; **p < 0.01; and ***p < 0.001 vs. the PBS group). (**c**) Distribution of bacteria in vivo.

The antitumor activity of EcNe/HlyE was investigated in tumor-bearing mice. The mice xenografted with either SW620 or HT29 cells were randomly divided into four groups (n = 5). Each group was administrated with EcNe or EcNe/HlyE while PBS was used as a control. As a result, there was no significant difference in body weights of mice that received any treatments (Fig. 5a). The administration of EcNe/HlyE readily caused tumor regression (Fig. 5b). The final tumor volume in mice bearing either SW620 or HT29 cells was reduced to 11 ± 4 and 369 ± 65 mm^3^, respectively. In contrast, tumors in control mice grew continuously. The volume of SW620 and HT29 tumors in the PBS-treated (control) group increased to 859 ± 70 and 1508 ± 56 mm^3^, respectively. As compared to the control, the administration of EcNe/HlyE reduced the tumor volume of SW620 cells and of HT29 cells by approximately 98% and 76%, respectively. Interestingly, EcNe without HlyE enabled to retard the growth of HT29 tumor. It suggests that SW620 tumor is more susceptible to HlyE than HT29 tumor. Moreover, the administration of EcNe/HlyE by intratumoral injection also reduced tumor volumes (SI Fig. S3). The result indicates that BCT based on EcNe/HlyE is not affected by the injection route.

The bacterial CFU count was implemented to determine the distribution of either EcNe or EcNe/HlyE in mice bearing SW620 or HT29 tumors. On the basis of per gram tissue, 10^8^–10^9^ CFU were found in the tumor and 10^2^–10^3^ CFU in the organ involving heart, liver, spleen, lung, and kidney (Fig. 5c). It clearly indicates preferential accumulation of EcNe and EcNe/HlyE in tumors.

### Histological morphology of tumor tissues

**Figure 6:**
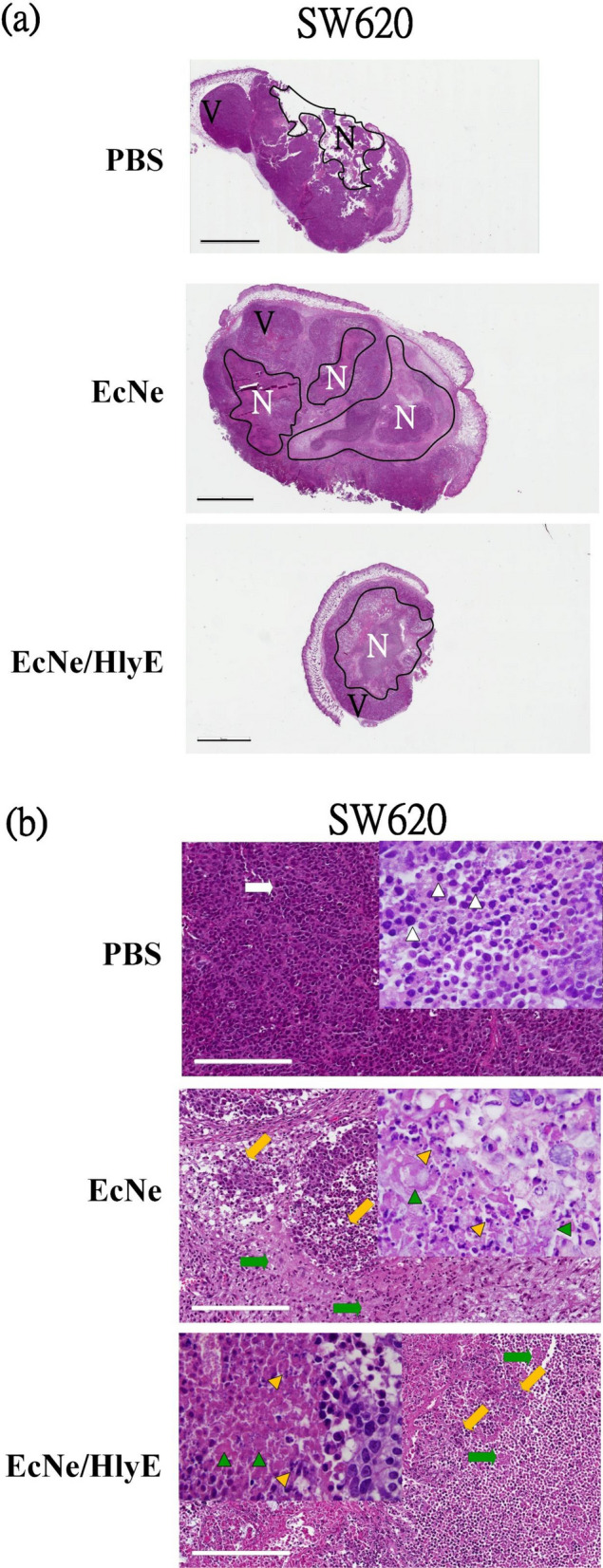

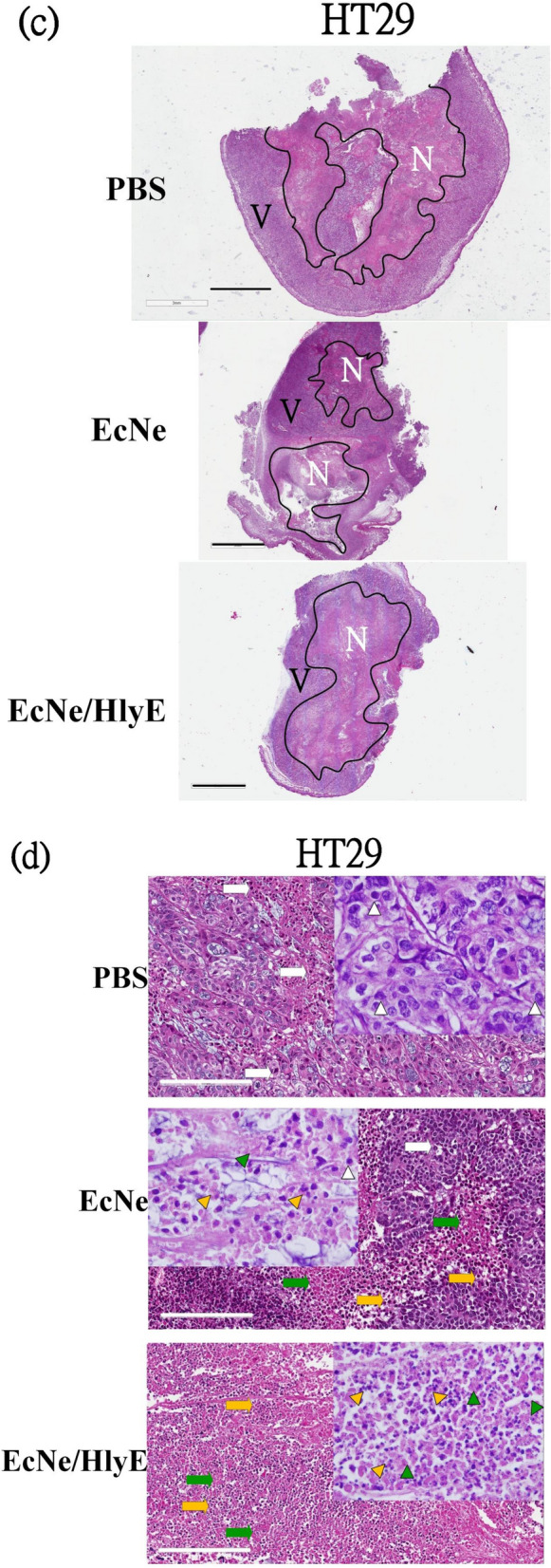

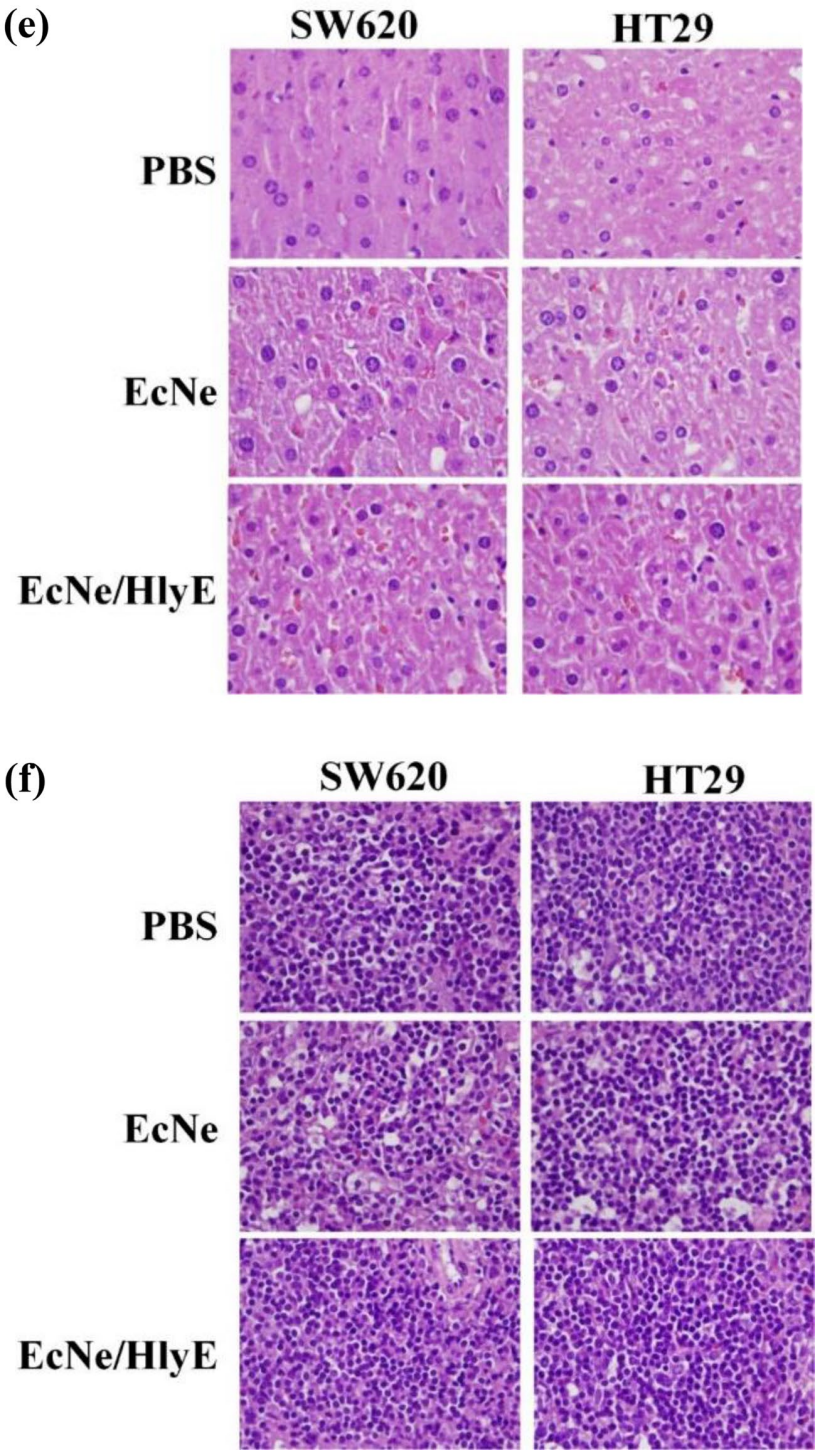
H&E staining of tumor tissues and organs**.** H&E staining was applied to analyze nuclear chromatin (purple-blue) and cytoplasm (red). (**a**) Tumor tissues of SW620 cells after receiving various treatments. The necrosis (N) and viable (V) regions of tissues were marked (bar = 3 mm). Imag J software was applied to measure the total cross-sectional area of each tumor and the area of necrosis present in each tumor. (**b**) High-resolution micrographs for necrosis regions of SW620 tissues (bar = 200 μm). Nuclear condensation (pyknosis), nuclear fragmentation (karyorrhexis), and nuclear dissolution (karyolysis) were observed as indicated by white, yellow, and green arrows, respectively. (**c**) Tumor tissues of HT29 cells after receiving various treatments. (**d**) High-resolution micrographs for necrosis regions of HT29 tissues. (**e**) Histopathological morphology of liver. (**f**) Histopathological morphology of spleen.

The morphological change in tumor tissues of mice was observed by the H&E staining. SW620 tumor tissues assumed a fairly complete structure and regular shapes. The administration with EcNe/HlyE increased the extent of necrosis, consequently leading to the reduction of tumor (region N in Fig. 6a,b). Necrosis was surrounded by viable tissue (region V). In contrast, the necrotic region was present in HT29 tumor tissues irrespective of the treatment (Fig. 6c,d). In general, the necrotic cell type of pkynosis was more common in the EcNe/HlyE-caused tumor necrosis. Cells with karyorrhexis and karyolysis also appeared in the necrotic region. The necrotic region was bounded by cells with karyorrhexis. Overall, the fraction of viable tissue was largely reduced after the treatment of EcNe/HlyE.

The histopathological study showed that the morphology of organs remained unaffected for all types of treatment (Fig. 6e,f). Liver and spleen were all free from damage.

### Investigation of immune responses

**Figure 7:**
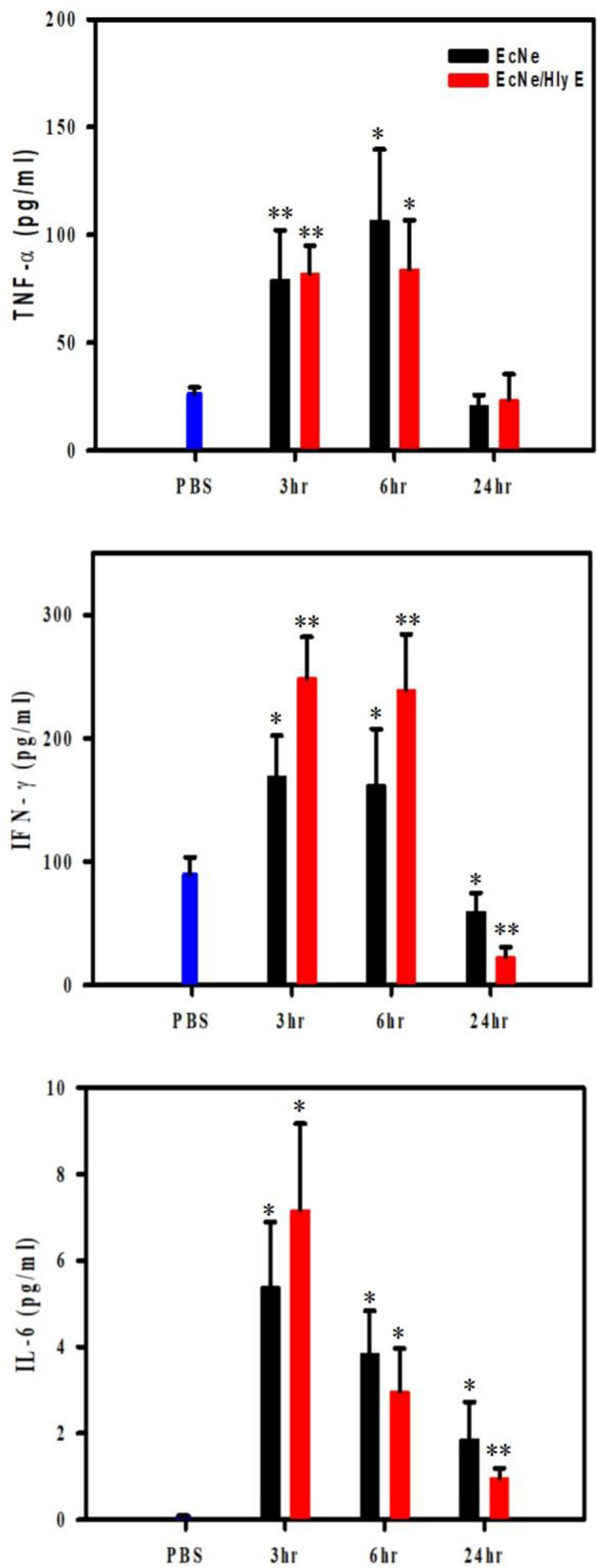
Investigation of immune responses in mice. C57BL/6 mice were treated with systemic administration of PBS, EcNe or EcNe/HlyE for four consecutive days. Since then, their serum levels of TNF-α, TNF-γ and IL6 in C57BL/6 mice were quantified at the indicated time three time points. Values were expressed as means ± SD (*p < 0.05; **p < 0.01; and ***p < 0.001 vs. the PBS group).

Finally, the safety issue of the HlyE-producing bacteria was further addressed by investigation of the immune response in mice. This was conducted with C57BL/6 mice that received two intravenous injections of HlyE and either EcNe or EcNe/HlyE. As a result, serum TNF-α, TNF-β and IL6 levels slightly increased in the first 6 h after the final injection. Their levels returned to nearly the basal level at 24 h (Fig. 7). The result indicates that the severe host immune response and the prolonged inflammatory cytokine response are not induced in EcNe/HlyE-treated animals.

## Discussion

The platform for BCT based on therapeutic proteins basically consists of a bacterial vector, a protein payload, and a protein-expressing system. The bacterial vector is mainly responsible for the synthesis and in situ delivery of the protein payload. In this context, the bacterial ability of seeking tumor turns out to be a perquisite. The potential of probiotic EcN for cancer therapy was first acknowledged by recognizing its high efficiency of 4T1 breast tumor-specific colonization and replication^38^. In particular, the result of tumor colonization was not affected by the administration routes involving intraperitoneal or intratumoral injection of EcN. In this study, EcN was developed by metabolic engineering for medical intervention of colorectal cancer. The engineered strain (i.e., EcNe) specifically adhered to SW620 and HT29 cancer cells in vitro (Fig. 4a) whereas cancer cells were free from detriment (Fig. 4b). EcN has an extracellular structure composed of K5 capsule^39^ and possesses F1C fimbriae for formation biofilm^40^, which confers on EcN the intestinal colonization ability. These characteristic structures of EcNe likely interact with these colorectal cancer cells. EcNe preferentially colonized in SW620 and HT29 tumors (Fig. 5c) while retarded the growth of HT29 tumor (Fig. 5b). It was reported that EcN displayed high efficiency of tumor-selective colonization and amplification in both immunocompetent and immunocompromised mice^38^. Therefore, the reduced growth rate of HT29 tumor by EcNe is likely attributed to a high density of colonized bacteria that competes for available nutrients.

A potential protein payload needs to be secreted from the bacterial vector, highly cytotoxic against malignant cancer cells, and capable of diffusing deep into tumor tissue. Recognized as a small hemolytic protein, HlyE is exported from *E. coli* via the outer-membrane vesicles (OMVs)-mediated transport route^41^. OMVs-released HlyE displayed a high cytotoxic activity toward mammalian cells. Accordingly, HlyE has been applied for cancer therapy. The administration of *S. typhimurium* which expressed HlyE with a hypoxia-inducible promoter significantly increased necrosis of 4T1 breast tumor^15^. In another study, the growth of 4T1 breast and CT26 murine colon tumors were marginally affected by *E. coli* strain K-12 with HlyE under the control of a constitutive promoter^42^. In this study, *E. coli* HlyE was produced by a P_*BAD*_–based expression system in EcNe. P_*BAD*_ is known to be stringently regulated and promptly responding to the induction of L-arabinose^33^. In addition, L-arabinose enables passive diffusion into tumor tissue and administration via the oral route. EcN was reprogrammed to improve the performance of the P_*BAD*_-based expression system, resulting in EcNe. EcN displayed a homogenous and glucose-insensitive induction profile and achieved a saturated production at a very low level of inducer. As shown in Fig. 2, EcNe/HlyE was far superior to EcN/HlyE in terms of the HlyE production. The production of P_*BAD*_-regulated HlyE in EcNe/HlyE is insensitive to glucose and saturated at 30–50 μM L-arabinose (indicative of bacterial population homogeneity). Consequently, EcNe/HlyE exhibited the tumor-specific colonization with the tumor-to-organ ratio of 10^6^:1 and significantly reduced the volume of SW620 tumor and HT29 tumor by 98% and 76% (Fig. 5b,c), respectively.

*E. coli* HlyE and *S. aureus* SAH share a similar function of pore-forming. The previous study reported that most of SAH regulated by P_*BAD*_ was released outside from *E. coli* strain K^17^. Mice were injected with L-arabinose (2.67 M), and the treatment with SAH-producing *E. coli* significantly reduced the volume of MCF7 breast tumor and increased the extent of cell necrosis. In contrast to the injection route, the oral administration of L-arabinose at 100 mM in this study was effective to induce the protein payload in mice. Note that *S. typhimurium* were cleared from the blood at 6 h and the liver and spleen at 48 h post injection^7^. Similarly, EcN were mostly found in tumors at 24 h after injection^38^. This is advantageous for a temporal control of the protein expression by P_*BAD*_, which enables constraint of the expressed payload to tumor. In their SAH study, a mathematical model was proposed and predicted the positive linkage of bacterial colony size and the protein production rate to SAH efficacy^17^. However, the colony size was found to increase with decreasing the production rate. The bacterial growth is usually afflicted by the protein overproduction, known as “metabolic burden”^43^. This physiological stress would exaggerate the characteristic heterogeneity of P_*BAD*_ as described earlier. Therefore, a large colony size mainly consists of uninduced bacteria due to their fitness for growth. The interference of the blood sugar level with P_*BAD*_ also negatively affects the protein production rate. Colonized bacteria survive in the transition zone between the proliferative and necrotic regions of tumors where the microenvironment constantly varies^44^. This further renders the spatial control of the protein expression by P_*BAD*_ complicate. Reprogrammed EcNe appears feasible to address the issue (see discussion earlier). As analyzed by time-lapse images, tumor cells readily displayed a characteristic morphological change of oncosis upon treatment with EcNe/HlyE (Fig. 4c,d). In tumor-bearing mice, colonized EcNe/HlyE maintained L-arabinose inside and continuously expressed HlyE with a sufficient level to kill tumor cells. The necrotic regions of tumors thus enlarged (Fig. 6a–d), and EcNe/HlyE which lived in the border regions between viable and necrotic tissues moved toward to the tumor peripheral. The tumor microenvironment changed with the dynamics of the bacteria-tumor cells interaction. Consequently, the tumor growth of HT29 tumor was arrested while SW620 tumor was nearly removed (Fig. 5b).

In contrast to previous studies conducted with murine tumor models^25,26^, this study investigated the cancer therapeutics of HlyE-producing EcN (i.e., EcNe/HlyE) in human tumor models. A study also reported that a fusion protein-expressing EcN exhibited efficient inhibition of the growth of human hepatoma SMMC-7721 tumor^45^. This hybrid protein consisted of the tumor suppressor protein p53 and the anti-angiogenic peptide Tum-5 under the control of the oxygen-dependent promoter from *Vitreoscilla*. Overall, the encouraging results from these various studies suggest the potential application of EcN for cancer therapy. As shown in Fig. 7, the induction of acute infection was absent from injection of EcNe/HlyE. By regulation of newly-recruited T cells, EcN was able to reduce intestinal inflammation^46^. Therefore, probiotic EcN manifests itself as an alternative to *Salmonella* that has been intensively investigated for conveyance of antitumor drugs^47^.

In summary, colorectal cancer is a life-threatening disease due to the failure of conventional therapies in many cases. In this study, a new method of bacteria-mediated tumor therapy was successfully developed by exploiting EcN for selective delivery of a small cytotoxic protein (HlyE). To approach the goal, EcN was finely tailored by metabolic engineering. The resulting EcNe enabled the temporal and spatial control of the P_*BAD*_-regulated HlyE expression to improve its therapeutic efficacy. Production of recombinant proteins has been most commonly practiced in *E. coli*. Likewise, a tune-up of EcN is feasible to achieve efficient production of the therapeutic protein. Metabolic engineering provides a powerful method for rational design of a bacterial strain with the desired trait. We have applied this strategy to improve the protein production and alleviate the stress of metabolic load in *E. coli*^48^. With availability of the synthetic biology toolbox^49^, these technology advances are expected to design a robust system based on EcN for medical intervention of various tumors.

## Supplementary Information

Supplementary Information
