# Supplementary material for: Metabolic engineering of probiotic Escherichia coli for cytolytic therapy of tumors
Source: Sci Rep. 2021 Mar 12;11:5853. doi: 10.1038/s41598-021-85372-6 (PMC7971005; doi:10.1038/s41598-021-85372-6)
Supplement: Supplementary file 1 — Supplementary Information [file 41598_2021_85372_MOESM1_ESM.pdf]

Metabolic engineering of probiotic *Escherichia coli* for cytolytic therapy of tumors

Chung-Jen Chiang,[†,*] Po-Han Huang[†]

[†]Department of Medical Laboratory Science and Biotechnology, China Medical University, No. 91, Hsueh-Shih Road, Taichung, Taiwan 40402

*Address correspondence to:

Dr. Chung-Jen Chiang

E-mail: cjchiang@mail.cmu.edu.tw

TEL: 886-4-22003366 ext. 7227

Fax: 886-4-22057414

Supplementary Dataset File

The SDS-PAGE analysis of HlyE in full-length gels.

Figure 2a (top). The SDS-PAGE analysis of HlyE.

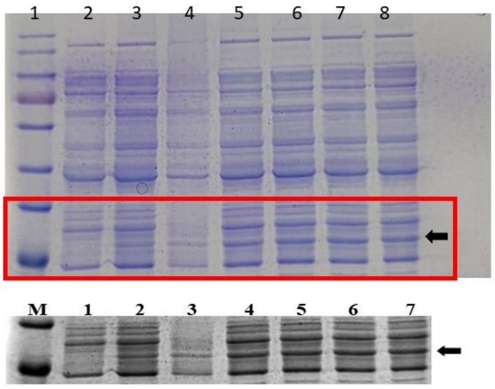

The production of HlyE in EcNe/HlyE (top) was conducted with LB medium.
1.Marker
2.EcN-e/pBAD33-hlyE
3.EcN-e /pBAD33-hlyE + 10 μM L-ara
4.EcN-e /pBAD33-hlyE + 30 μM L-ara
5.EcN-e /pBAD33-hlyE + 50 μM L-ara
6.EcN-e /pBAD33-hlyE + 100 μM L-ara
7.EcN-e /pBAD33-hlyE + 1 mM L-ara
8.EcN-e /pBAD33-hlyE + 10 mM L-ara

Figure 2a(bottom). The SDS-PAGE analysis of HlyE.

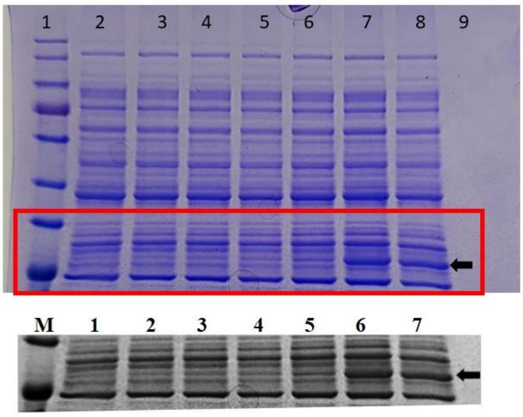

The production of HlyE in EcN/HlyE (bottom) was conducted with LB medium.
1.Marker
2.EcN/pBAD33-hlyE
3.EcN/pBAD33-hlyE + 10 μM L-ara
4.EcN/pBAD33-hlyE + 30 μM L-ara
5.EcN/pBAD33-hlyE + 50 μM L-ara
6.EcN/pBAD33-hlyE + 100 μM L-ara
7.EcN/pBAD33-hlyE + 1 mM L-ara
8.EcN /pBAD33-hlyE + 10 mM L-ara

The SDS-PAGE analysis of HlyE in full-length gels.

Figure 2b. The effect of glucose on the HlyE production in EcN-e /pBAD33-hlyE

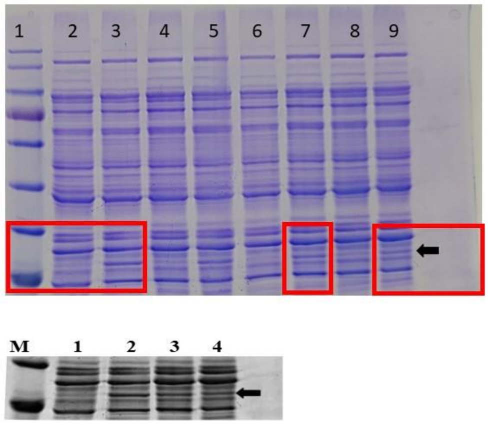

Bacteria were grown on LB medium plus various amounts of glucose. The HlyE production was induced in EcNe/HlyE and by adding 50 μM L-arabinose.

1.Marker
2.LB
3.LB + 50 μM L-ara
4.LB + 1% glc
5.LB + 1% glc + 50 μM L-ara
6.LB + 4% glc
7.LB + 4% glc + 50 μM L-ara
8.LB + 10% glc
9.LB + 10% glc + 50 μM L-ara

Figure 2c(top).
The effect of glucose on the HlyE production in EcN/pBAD33-hlyE

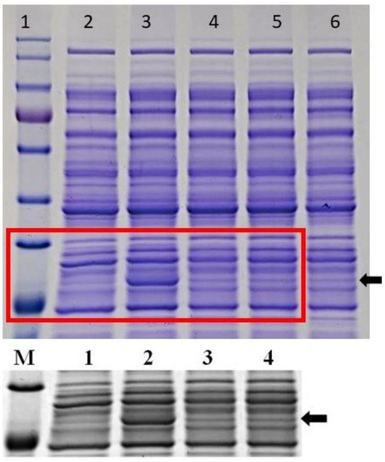

Bacteria were grown on LB medium plus various amounts of glucose. The HlyE production was induced in EcN/HlyE by adding 1 mM L-arabinose.

1.Marker
2.LB
3.LB + 1 mM L-ara
4.LB + 1% glc + 1 mM L-ara
5.LB + 4% glc + 1 mM L-ara
6.LB + 10% glc + 1 mM L-ara

Figure 2c(bottom).
The effect of glucose on the HlyE production in EcN/pBAD33-hlyE

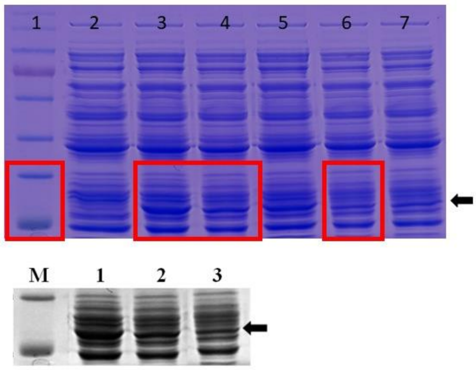

Bacteria were grown on LB medium plus various amounts of glucose. The HlyE production was induced in EcN/HlyE by adding 1 mM L-arabinose.

1.Marker
2.LB
3.LB + 1 mM L-ara
4.LB + 0.1% glc + 1 mM L-ara
5.LB + 0.1% glc + 10 mM L-ara
6.LB + 0.2% glc + 1 mM L-ara
7.LB + 0.2% glc + 10 mM L-ara

Supplementary Information

Materials and methods

Haemolysis assay

Healthy female C57BL/6 mice (n=5) with the age of 6 to 8 weeks old were purchased from the National Laboratory Animal Center, Taiwan. Blood samples were freshly collected in a centrifuge tube containing 50 mL EDTA and centrifuged at 2500 rpm for 5 min. Red blood cells (RBCs) were separated from plasma and washed with 1x PBS for 3 times. The number of RBCs suspended in 1x PBS was counted by using a hemacytometer.

Various amounts of HlyE were added to RBCs (2×10^6 /ml) and mixed gently. Samples were kept at 37°C for 30 min and centrifuged at 3,000 rpm for 3 min. Supernatant (100 μl) was transferred to microwell plate and analyzed by using ELSA plate reader with the absorbance at 570 nm. Water and PBS served as the positive and negative control, respectively. The percentage of RBCs haemolysis was calculated in the following:

Percentage of RBCs haemolys is

$$= \frac{\text{Sample absorbance} - \text{negative control absorbance}}{\text{positive control absorbance} - \text{negative control absorbance}} \times 100$$

The tryptic soy agar plates containing 5% sheep blood was used to investigate the hemolytic activity of HlyE. The protein samples (20 μl) from non-induced and induced EcNe/HlyE were spotted on sheep blood agar plates. The HlyE dose of 1 μg was administrated. A hemolytic zone around the hole was observed after plates were incubated at 37°C overnight.

(a)

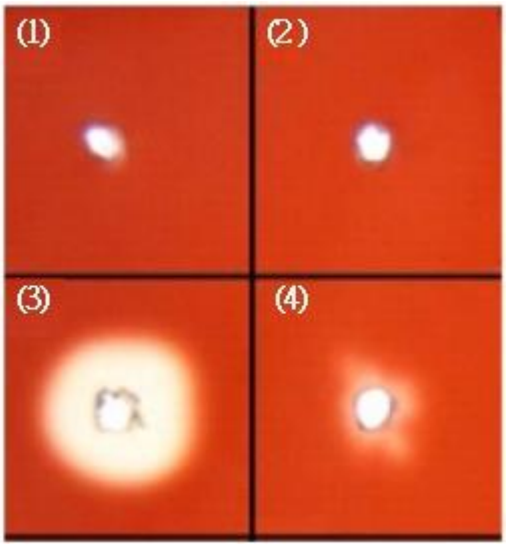

(b)

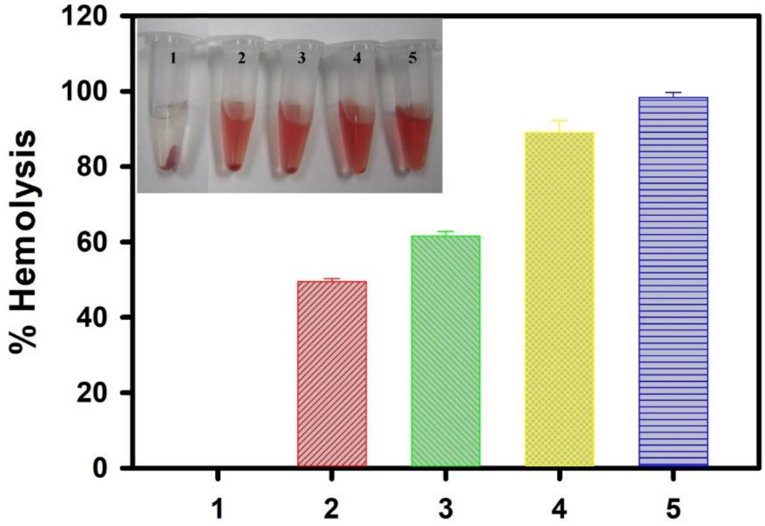

Fig. S1. HlyE production and functional activity. (a) Detection of the HlyE activity on sheep blood agar plates. Keys: 1, intracellular HlyE from EcNe/HlyE; 2, PBS, 3, intracellular HlyE from induced EcNe/HlyE; 4, extracellular HlyE from induced EcNe/HlyE. (b) The hemolysis of RBCs by HlyE. RBCs were incubated with various levels of HlyE from induced EcNe/HlyE for 30 min. Keys: 1, Mock; 2, 0.85 μg/ml; 3, 1.7 μg/ml; 4, 8.5 μg/ml, 5: ddH_2O.

SW620

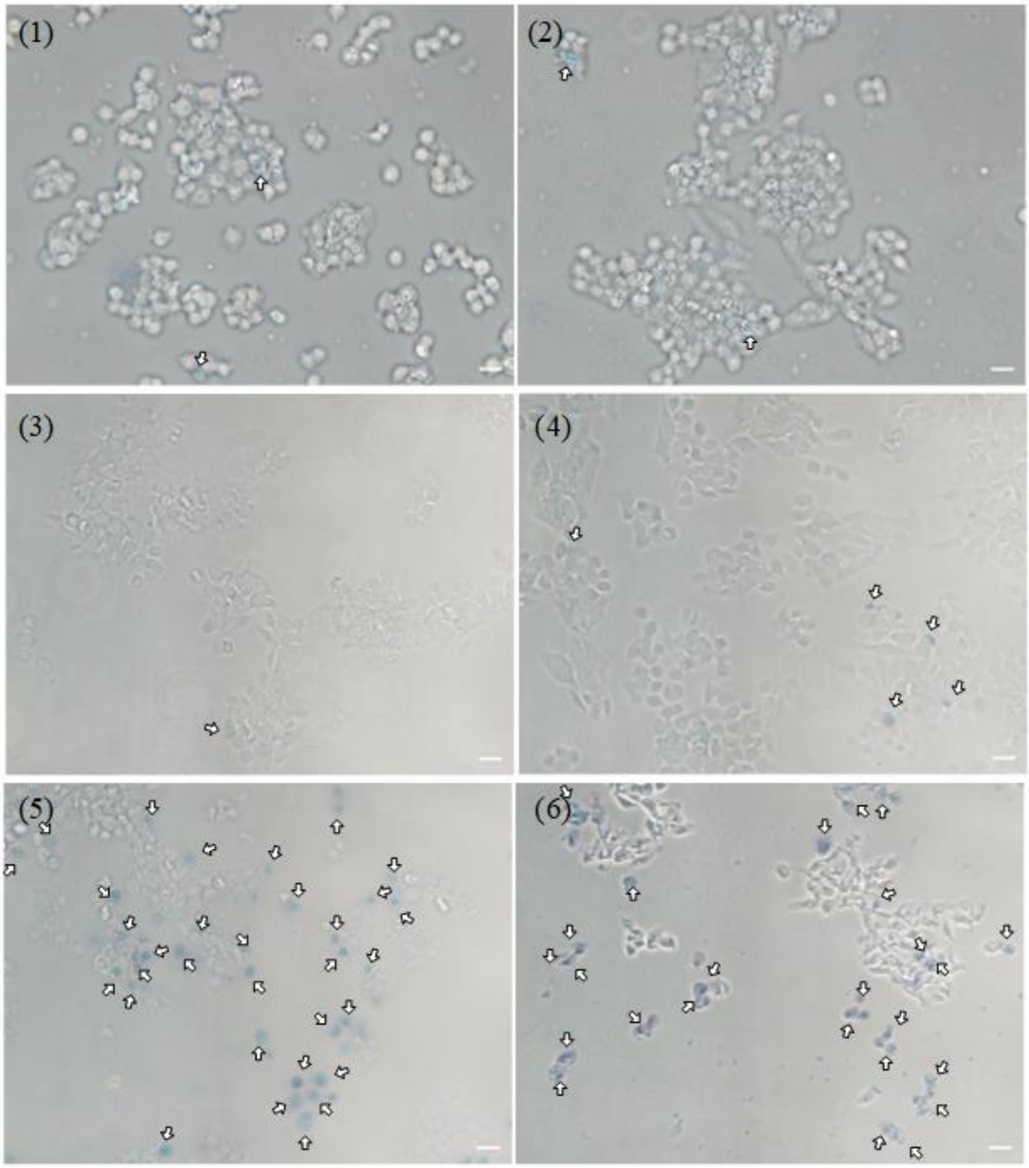

HT29

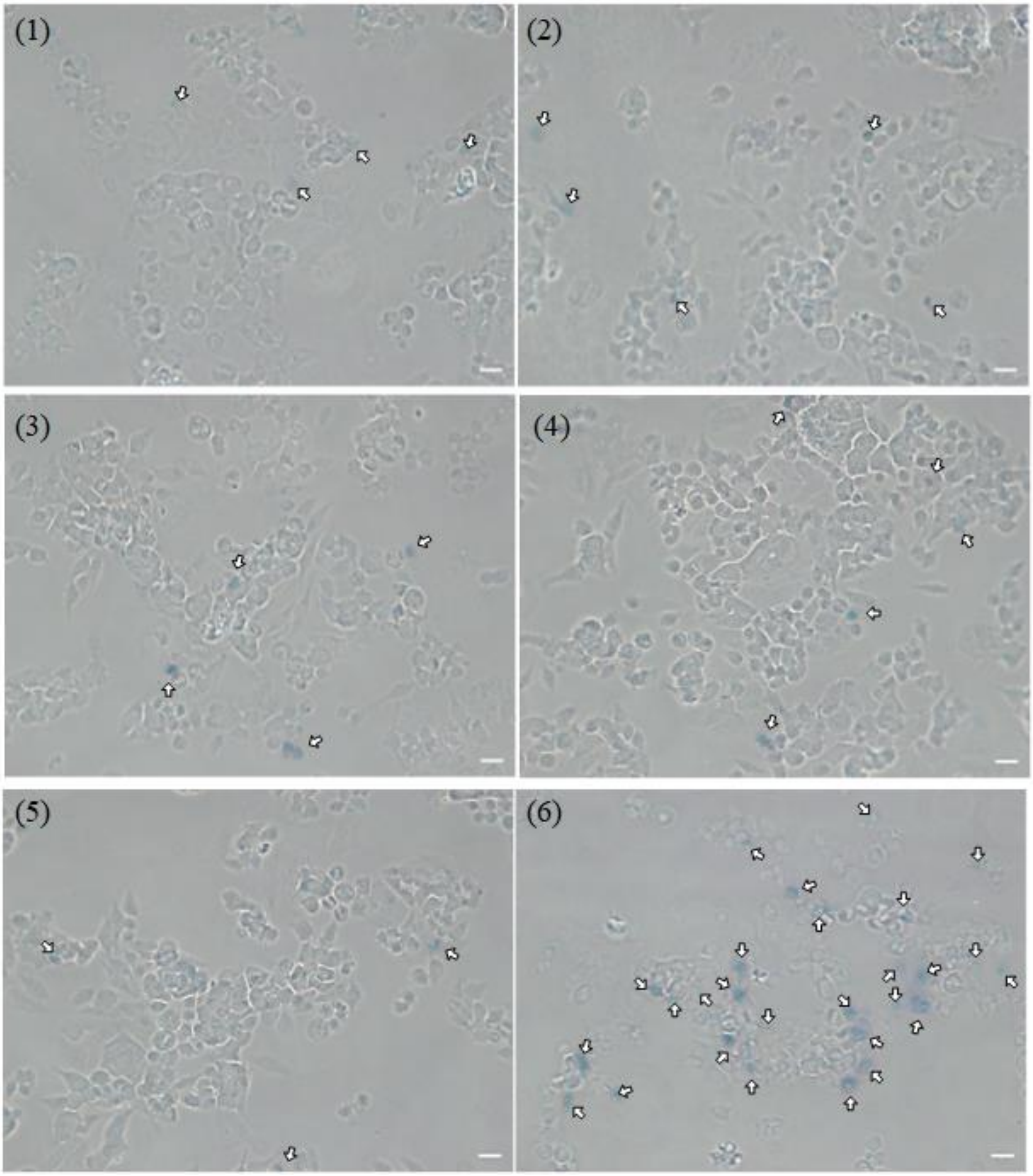

Fig S2. Examination of cell viability by trypan blue exclusion assay. EcNe which harbored plasmid pBAD33 (i.e., EcNe/pBAD33) was used as a control. After the treatment for 30 min, cell suspension was mixed with an equal amount of 0.4% trypan blue dye. The mixture was incubated 2 min at room temperature. Keys: 1, Mock; 2, LB medium; 3, culture medium from induced EcNe/pBAD33; supernatant from induced EcNe/HlyE; 5, extracellular HlyE from induced EcNe/HlyE; 6, intracellular HlyE from induced EcNe/HlyE.

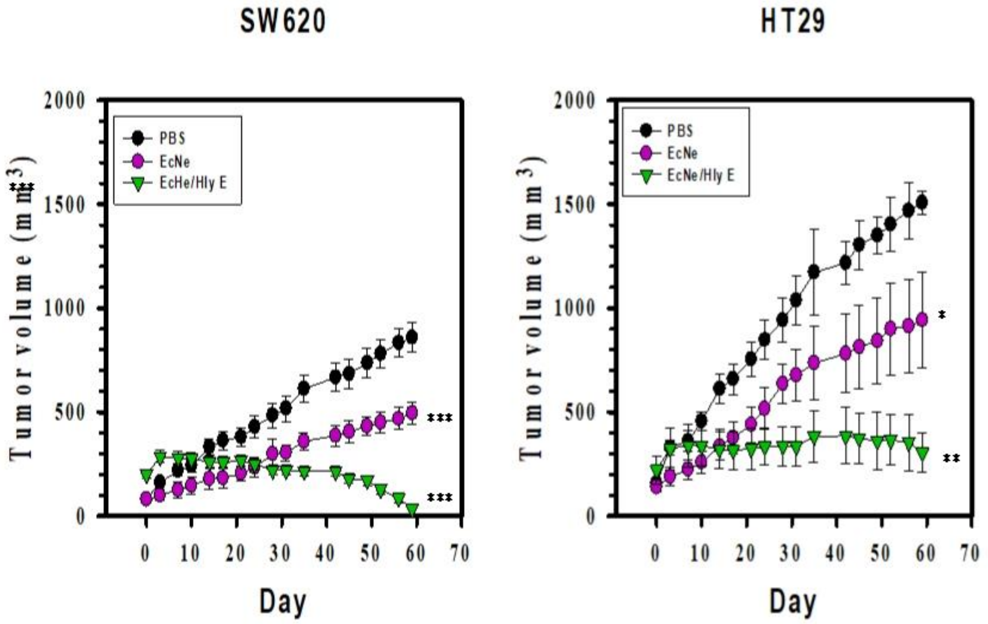

Fig. S3. Tumor regression by intratumoral injection of EcNe/HlyE. (a) The change in the body weight of mice receiving the treatment. (b) Therapeutic effect of EcNe/HlyE on mice (n=5) bearing SW620 or HT29 tumor cells. Tumor volumes (mm^3) were estimated using external calipers, and values were expressed as means ± SD (*p<0.05; **p<0.01; and *** p<0.001 *vs*. the PBS group).
